# Role of *Trachemys scripta elegans* in polystome (Platyhelminthes, Monogenea, Polystomatidae) spillover and spillback following the trade of freshwater turtles in southern Europe and North America

**DOI:** 10.1051/parasite/2025022

**Published:** 2025-05-21

**Authors:** Olivier Verneau, Dennis Quinn, Kevin G. Smith, John H. Malone, Louis du Preez

**Affiliations:** 1 Université Perpignan Via Domitia, Centre de Formation et de Recherche sur les Environnements Méditerranéens, UMR 5110 66860 Perpignan France; 2 CNRS, Centre de Formation et de Recherche sur les Environnements Méditerranéens, UMR5110 66860 Perpignan France; 3 Unit for Environmental Sciences and Management, North-West University Potchefstroom 2531 South Africa; 4 CTHerpConsultant 40 Pine Street Plantsville CT 06479 USA; 5 Davidson College, Biology Department Box 7118 Davidson NC 28035 USA; 6 Institute of Systems Genomics, Department of Molecular and Cell Biology, University of Connecticut Storrs CT 06269 USA; 7 South African Institute for Aquatic Biodiversity Somerset Street Makhanda 6139 South Africa

**Keywords:** *Trachemys scripta elegans*, Parasites, Polystomes, Invasions, Reservoir host, Spillover event, Spillback event

## Abstract

The red-eared slider, *Trachemys scripta elegans* (Wied, 1938), has been introduced worldwide, partly because of the exotic pet trade in the 1980s and 1990s. When *T. s. elegans* is released or escapes into natural environments, it often establishes new feral populations due to its tolerance for a variety of aquatic ecosystems. Therefore, it is now considered one of the most invasive species in the world because it can compete with native turtle species. In the present study, our objectives were to identify the potential for polystome spillover and spillback resulting from the introduction of the red-eared slider into new environments in North America. Fieldwork investigations were thus conducted mainly in aquatic habitats in Florida and North Carolina, United States, but also in Connecticut, Indiana, Kansas, Maine, Nebraska and New York. Using DNA barcoding based on cytochrome c oxidase I (COI) sequences, we surveyed the species diversity of polystome within American freshwater turtles. These included *T. s. elegans* but also *Apalone ferox*, *Apalone spinifera*, *Chelydra serpentina*, *Chrysemys picta*, *Kinosternon baurii*, *Pseudemys* spp., *Sternotherus minor* and *Sternotherus odoratus*. Genetic evidence confirmed that invasive populations of *T. s. elegans* in southern Europe have transmitted their own polystomes to native host species following spillover effects, and revealed here that *T. s. elegans* in non-indigenous habitats in the United States acts as a new reservoir of infection for native polystomes following spillback effects, thus increasing indigenous parasite transmission in the wild. Together, these findings raise further concern about the spread of non-native turtles and their impact on parasite transmission.

## Abstract (fr)

La tortue à tempes rouges, *Trachemys scripta elegans* (Wied, 1938), a été introduite dans le monde entier, en partie grâce au commerce d’animaux de compagnie exotiques dans les années 1980 et 1990. Lorsque *T. s. elegans* est relâchée ou s’échappe dans des milieux naturels, elle établit souvent de nouvelles populations sauvages suite à sa tolérance à une variété d’écosystèmes aquatiques. De ce fait, elle est aujourd’hui considérée comme l’une des espèces les plus invasives au monde, car elle peut concurrencer les espèces de tortues indigènes. Dans cette étude, nos objectifs étaient d’identifier le potentiel de propagation et de transmission des polystomes résultant de l’introduction de la tortue de Floride à tempes rouges dans de nouveaux environnements en Amérique du Nord. Des campagnes de terrain ont donc été menées principalement dans les habitats aquatiques de Floride et de Caroline du Nord aux États-Unis, mais aussi dans le Connecticut, l’Indiana, le Kansas, le Maine, le Nebraska et l’État de New York. En utilisant le code-barre ADN basé sur les séquences de la cytochrome c oxydase I (COI), nous avons étudié la diversité des espèces de polystomes chez les tortues d’eau douce américaines. Celles-ci comprenaient *T. s. elegans* mais aussi *Apalone ferox*, *Apalone spinifera*, *Chelydra serpentina*, *Chrysemys picta*, *Kinosternon baurii*, *Pseudemys* spp., *Sternotherus minor* et *Sternotherus odoratus*. Les preuves génétiques ont confirmé que les populations invasives de *T. s. elegans* en Europe du Sud ont transmis leurs propres polystomes aux espèces hôtes indigènes suite à des effets de débordement, et ont révélé ici que *T. s. elegans* dans les habitats non indigènes des États-Unis agit comme un nouveau réservoir d’infection pour les polystomes indigènes suite à des effets boule de neige, augmentant ainsi la transmission des parasites indigènes dans la nature. Ces résultats suscitent de nouvelles inquiétudes quant à la propagation des tortues non indigènes et à leur impact sur la transmission du parasite.

## Introduction

Vector-borne diseases of humans, livestock and wildlife are transmitted by diverse invertebrate hosts, including mosquitoes, sandflies, ticks, flies, fleas, lice and aquatic snails [16, 46]. Vectors typically act as carrier hosts where pathogens multiply and develop before being transmitted to another definitive or intermediate host, mostly actively through direct contact although other processes are sometimes considered [53]. Besides the significant role played by vectors in disease transmission, numerous animals comprising fish, amphibians, reptiles, birds and mammals can also act as disease reservoirs for transmitting parasites to other animals [21]. If reservoir hosts serve as healthy carriers of parasites, once transmitted to other host competent species, parasites can potentially cause significant zoonotic health issues. This was illustrated in numerous cases following handling of wild animals. For example, it is now well known that salmonellosis is associated with exotic pets, especially iguanas and turtles [52, 54]. Between 1996 and 1997 in the United States, there were 74,000 *Salmonella* infections that were associated with reptile or amphibian contact [34], and strong correlations were observed between salmonellosis and turtle handling during *Salmonella* outbreaks [19, 20]. Whereas the presence of *Salmonella* has been detected in free living native turtles across natural wetland environments of Southwestern Europe, it was also documented in the same environments from feral populations of the common slider *Trachemys scripta* (Thunberg, 1792), as well as from pet turtles. This demonstrates that feral populations of *T. scripta* acting as animal reservoirs may represent an additional risk factor for *Salmonella* or some other parasite infections for humans and native animals [24, 33]. This is why host–parasite interactions involving *T. scripta* should be investigated more in depth in the environments where sliders were introduced.

With just over 200 species reported, polystomes (Platyhelminthes, Monogenea, Polystomatidae) infect semi-aquatic vertebrates, including the Australian lungfish *Neoceratodus forsteri* (Krefft, 1870), amphibians, freshwater turtles and the common hippopotamus, *Hippopotamus amphibius* Linnaeus, 1758 [12]. These parasites, which are globally distributed, are recovered mostly from the gills and/or the urinary bladder of amphibians, and from the urinary bladder, pharyngeal cavity, or conjunctival sacs of freshwater turtles, while they are reported from gills and skin of lungfishes and from conjunctival sacs of hippopotamuses. They all display a direct life cycle with no intermediate host, which involves mature parasites that produce and release eggs at different rates depending of the type of host, host reproductive status, host behaviour and external temperature (see Du Preez *et al.* [12]). Once eggs are released into the water, ciliated larvae develop and hatch usually within two to three weeks. After contact with a suitable host, larvae use different migration routes on the host depending on the polystome genus. Larvae of *Apaloneotrema*, *Aussietrema* and *Fornixtrema* migrate to the conjunctival sacs, while larvae of *Polystomoidella* and *Uropolystomoides* enter *via* the cloaca before establishing in the urinary bladder. Lastly, larvae of *Manotrema* and *Uteropolystomoides* establish directly in the mouth and pharyngeal pouches (see Du Preez *et al.* [12]). Because *Pleurodirotrema* and *Polystomoides* both include species that infect either the urinary bladder or the oral region of their hosts [5, 10], they complete the two distinct life cycles as described above depending on the parasite’s ecological niche. While polystomes can be regarded mostly as host- and site-specific parasites (see Du Preez *et al.* [12] for a review), some polystomes that infect turtles have also been reported from several host species living in outdoor turtle enclosures at zoological aquariums or gardens, turtle farms or private properties [51]. For example, this is the case for *Polystomoides coronatus* (Leidy, 1888 [30]), which was originally described from the pharyngeal cavity of an unidentified American turtle. According to Price [41], this parasite infected the urinary bladder of the red-eared slider *Trachemys scripta elegans* (Wied, 1838) and the pharyngeal cavity of the spiny softshell turtle *Apalone spinifera* (Lesueur, 1827) at the zoological aquarium of New York City, NY. This is also the case for *Polystomoides orbicularis* (Stunkard, 1916 [43]), which was formerly described from the urinary bladder of the painted turtle *Chrysemys picta* (Schneider, 1783). This parasite was also reported to infect the pharyngeal cavity of the Florida softshell turtle *Apalone ferox* (Schneider, 1783) and the urinary bladder of the Cumberland slider *Trachemys scripta troosti* (Holbrook, 1836), *T. s. elegans* and *C. picta* at the zoological aquarium of New York City [41]. In the early 2010s, *Polystomoides oris* Paul, 1938 [36], which was originally described from the pharyngeal cavity of *C. picta* in aquatic ecosystems in the United States, and *P. orbicularis* were both recorded from European turtles including the Mediterranean pond turtle *Mauremys leprosa* (Schweigger, 1812) and the European pond turtle *Emys orbicularis* (Linnaeus, 1758) in outdoor turtle enclosures in southern France [51]. Overall, these results suggested that polystomes of turtles were not as strictly host-specific as originally hypothesized, at least in zoological parks where turtles can be found in the same artificial pools.

These conclusions were further supported by studies of polystome biodiversity within populations of native freshwater turtles in natural aquatic environments across Europe. With the exception of the native polystome species, namely *Polystomoides euzeti* (Combes & Ktari, 1976 [7]) and *Polystomoides tunisiensis* Gonzales & Mishra 1977 [17], which infect the urinary bladder and the pharyngeal cavity of *M. leprosa*, respectively, and *Polystomoides ocellatus* (Rudolphi, 1819 [42]), which is found in the pharyngeal cavity of *E. orbicularis*, all other polystomes found within both European turtles originated from North American turtles [22, 35]. Because non-native polystomes were also found within feral populations of *T. s. elegans* across European wetlands, it was concluded that these turtles may carry alien polystomes in southern Europe [22, 35] and transmit their parasites to indigenous turtles following spillover events.

**Figure 1:**
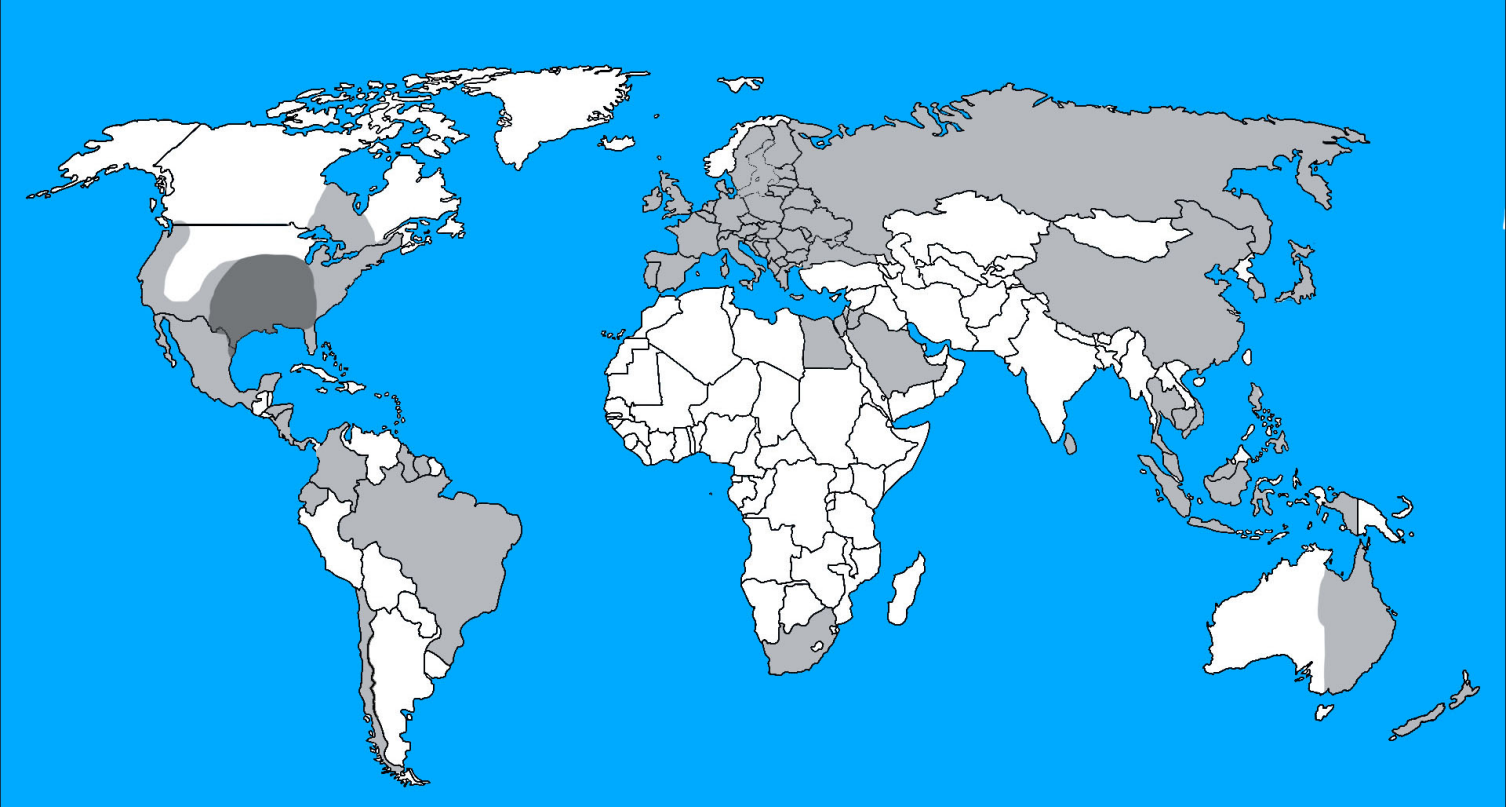
Schematic representation of the *T. s. elegans* range distribution according to Van Dijk *et al.* [50]. Original range in dark grey; range following human translocation in light grey.

These results thus raised important questions about the role of *T. s. elegans* as a disease reservoir following spillover or spillback events. These events are thought to account for non-indigenous parasite dispersal and indigenous parasite dynamics, respectively, following introduction of non-native host species into new environments [8, 9, 21, 28, 40]. In other words, the introduced host acts as a reservoir of infection for non-native parasites in spillover, whereas it acts as a new reservoir of infection for native parasites in spillback [28]. Therefore, to address the role of the red-eared slider in polystome dispersal and transmission, we studied the polystome diversity among turtles in American freshwater ecosystems, with a particular focus on *T. s. elegans*. Because the red-eared slider was also introduced to several regions of the United States outside its native range [50] (Fig. 1), we expected to find greater species richness of polystomes within native freshwater turtles in America in areas where they now co-occur with *T. s. elegans*. Cytochrome c oxidase I (COI) sequences used as DNA barcodes were then obtained from polystome eggs and/or specimens that were collected from several species of turtles during three decades of fieldwork along aquatic ecosystems in the United States (Connecticut, Florida, Indiana, Kansas, Maine, Nebraska, New York and North Carolina). The biodiversity of polystomes that was inferred from the survey of *T. s. elegans* in its native as well as in non-native areas of American and non-American freshwater environments was compared to that of other sympatric, or even syntopic, turtle species. Turtles concerned *A. ferox*, *A. spinifera*, the common snapping turtle *Chelydra serpentina* (Linnaeus, 1758), *C. picta*, the false map turtle *Graptemys pseudogeographica* (Gray, 1831), the striped mud turtle *Kinosternon baurii* (Garman, 1891), the eastern river cooter *Pseudemys concinna* (Le Conte, 1830), the Florida cooter *Pseudemys floridana* (Le Conte, 1830), the Florida redbelly turtle *Pseudemys nelsoni* Carr, 1938, the peninsula cooter *Pseudemys peninsularis* Carr, 1938, the loggerhead turtle *Sternotherus minor* (Agassiz, 1857) and the common musk turtle *Sternotherus odoratus* (Latreille, 1802) from North America, but also *M. leprosa* and *E. orbicularis* from Europe. Our results reinforce prior observations that *T. s. elegans* acts as a reservoir host for spillover of polystomes outside the United States. They shed further light on a parasite spillback effect by *T. s. elegans,* which could have significant consequences for native turtles in America and the dynamics of aquatic ecosystems worldwide.

## Materials and methods

### Ethics

All turtles mentioned in this study were examined taking into account local laws and regulations, and non-invasive techniques were used as much as possible to obtain the parasites without killing the hosts. Ethical clearance was obtained from the North-West University Animal Care ethics committee (Ethical clearance no. NWU-00256-17A5). Permits were obtained from the North Carolina Wildlife Resources Commission and the Connecticut Department of Energy & Environmental Protection for collection in 2015 (Permit numbers 15-SC01038, 18-SC01287 & 1515004).

### Areas of investigation

In the United States, *T. scripta* (*T. s. elegans* and *T. s. scripta*) was collected from freshwater ecosystems of Indiana and Kansas where it occurs naturally without human intervention, and in Florida, Maine and North Carolina where it was introduced. *Chrysemys picta* was sampled in Connecticut, Indiana, New York and North Carolina, *C. serpentina* in Nebraska and North Carolina, *A. ferox* and *Pseudemys* spp. in Florida and *A. spinifera* with *G. pseudogeographica* in Indiana. In Europe, *T. s. elegans*, *M. leprosa* and *E. orbicularis* were surveyed mainly in the southwestern and southeastern parts of France and in the northern part of Spain. *Mauremys leprosa* and *E. orbicularis* were also surveyed in Morocco and Algeria, as *T. s. elegans* does not occur there. Some turtles were also inspected for parasites in non-natural aquatic environments, especially through outdoor turtle enclosures of the turtle farm of Sorède in southern France, and to a lesser extent through outdoor turtle enclosures of two private properties in France, namely at La Sauzière-Saint-Jean and Béziers [51]. Turtles collected in artificial ornamental ponds and settling basins for wastewater were also examined.

### Host and polystome sampling

Fieldwork investigations were conducted towards the end of the 1990s to 2018, usually from early spring to end of summer in freshwater aquatic environments. Sites of interest were ponds, lakes and rivers (Table 1). Turtles were captured with traps that were baited with fish or pork liver, set in water bodies for one to several days and checked daily for the presence of turtles [22, 23, 35]. Turtles were also collected by hand or with the help of landing nets in outdoor turtle enclosures. After capture, turtles were individually placed in plastic containers with clean water to cover about half the turtle’s body for one to three days. Water was then filtered every day through a pair of soil sieves with mesh size 500 and 100 micron, respectively [51], and examined for the presence of polystome eggs. Collected eggs were preserved in 70% molecular grade ethanol for later molecular analysis. Worms were also collected following the dissection of several infected turtles when it was not feasible to extract adult polystomes without killing animals or simply to identify the location of parasites in the host. Some specimens were preserved in 10% buffered formalin for further biometric and morphological analyses, while others were stored in 70% molecular grade ethanol for DNA barcoding.

**Table 1:** Geographical areas of American and French aquatic environments investigated for turtles and their polystomes.

Country	Location	Name	GPS coordinates
USA: Connecticut	loc1Co	Bever pond	41.801066, −72.090027
USA: Connecticut	loc2Co	Railway pond	41.800764, −72.088475
USA: Florida	loc1Fl	Quail Heights golf course	30.166279, −82.673098
USA: Florida	loc2Fl	Ichetucknee River	29.975000, −82.758888
USA: Florida	loc3Fl	Ichetucknee Bridge	29.952778, −82.785833
USA: Florida	loc4Fl	Hornsby Spring_1	29.854722, −82.603333
USA: Florida	loc5Fl	Hornsby Spring_2	29.850280, −82.593300
USA: Florida	loc6Fl	Santa Fe River	29.833525, −82.679350
USA: Florida	loc7Fl	Deer Run Gainesville	29.716736, −82.297859
USA: Florida	loc8Fl	Gainesville	29.7, −82.3
USA: Florida	loc9Fl	Gainesville pond	29.670146, −82.401368
USA: Florida	loc10Fl	Santa Fe College campus pond 3	29.683781, −82.434618
USA: Florida	loc11Fl	Santa Fe College campus pond 1	29.683781, −82.434605
USA: Florida	loc12Fl	Santa Fe College campus bullfrog pond	29.682741, −82.438475
USA: Florida	loc13Fl	Gainesville pond	29.670146, −82.401368
USA: Florida	loc14Fl	USGS facility, pond 11 at Gainesville	29.725278, −82.417778
USA: Florida	loc15Fl	Deer Run Retention pond 1	29.716966, −82.397777
USA: Florida	loc16Fl	Deer Run Retention pond 2	29.708286, −82.399870
USA: Florida	loc17Fl	Spanish Spring	28.943611, −81.950833
USA: Indiana	Unknown		
USA: Kansas	Unknown		
USA: Maine	Unknown		
USA: Nebraska	Unknown		
USA: New York	loc1NY	Barret’s pond	41.463769, −73.923297
USA: North Carolina	loc1NC	Mooresville golf course	35.575493, −80.835494
USA: North Carolina	loc2NC	Norman’s small pond	35.570824, −80.848196
USA: North Carolina	loc3NC	Lake Norman Mooresville 1	35.570090, −80.856500
USA: North Carolina	loc4NC	Big Griffith pond	35.502222, −80.854527
USA: North Carolina	loc5NC	Small Griffith pond	35.501638, −80.855027
USA: North Carolina	loc6NC	Fountain twin pond	35.499194, −80.863194
USA: North Carolina	loc7NC	River Run Country Club, pond 1	35.467388, −80.805055
USA: North Carolina	loc8NC	River Run Country Club, pond 2	35.461805, −80.802833
USA: North Carolina	loc9NC	Beaty Road pond	35.508111, −80.845416
USA: North Carolina	loc10NC	Lake Norman Mooresville 2	35.495278, −80.860555
USA: North Carolina	loc11NC	River Run Estate	35.468361, −80.797972
USA: North Carolina	loc12NC	Regie’s pond in Hopewell	35.467055, −80.816027
USA: North Carolina	loc13NC	Horse Farm pond	35.459583, −80.796361
France	loc1Fc	Ponds of the Natural Regional Park of La Brenne (Rosnay)	46.722994, 1.169983
France	loc2Fc	Pond of Grands Georgats (Lucenay-lès-Aix)	46.691838, 3.496725
France	loc3Fc	Outdoor turtle enclosures (La Sauzière-Saint-Jean)	43.968036, 1.628861
France	loc4Fc	Ornamental pond (Barbotan-les-Thermes)	43.951083, −0.042583
France	loc5Fc	Settling basin for wastewater (Manduel)	43.822775, 4.463111
France	loc6Fc	Outdoor turtle enclosures (Béziers)	43.802630, 2.976869
France	loc7Fc	Pond of Soulès (Cravencères)	43.771933, 0.026741
France	loc8Fc	Ponds of Roquet (Saint-Gély-du-Fesc)	43.705194, 3.7942
France	loc9Fc	Pond of En Cassagne (Ordan-Larroque)	43.672708, 0.469852
France	loc10Fc	Lake of Salagou (Clermont-l’Hérault)	43.645177, 3.364486
France	loc11Fc	Pond of Or (Mauguio)	43.580644, 4.01565
France	loc12Fc	Lake Méjean (Lattes)	43.541075, 3.913544
France	loc13Fc	Reserve of Estagnol (Villeneuve-lès-Maguelone)	43.533025, 3.837041
France	loc14Fc	Wetlands of La Tour du Valat (Arles)	43.509438, 4.668858
France	loc15Fc	Reserve of Bagnas (Agde)	43.320666, 3.502111
France	loc16Fc	Pond of the pine forest (Port Leucate)	42.842228, 3.037011
France	loc17Fc	Small canal close of the lagoon of Salses-Leucate (Saint-Hippolyte)	42.802630, 2.976869
France	loc18Fc	Agly River	42.772744, 2.890466
France	loc19Fc	Têt River	42.715119, 2.934022
France	loc20Fc	Fosseille River	42.666922, 2.966888
France	loc21Fc	Tech River	42.539180, 2.861144
France	loc22Fc	Outdoor turtle enclosures (Sorède)	42.515275, 2.957716
France	loc23Fc	Baillaury River	42.462647, 3.090836
France	loc24Fc	Pond of Gradugine (Prunelli-di-Fiumorbo)	41.981591, 9.430697
France	loc25Fc	Rizzanese River (Propriano)	41.661919, 8.881963
France	loc26Fc	Rizzanese River (Portigliolo)	41.645461, 8.870211

### Molecular and phylogenetic analyses

DNA was extracted from eggs and/or adult worms following the procedure described in Verneau *et al.* [51]. Partial COI was amplified using the combination of either Forward L-CO1p (5′–TTTTTTGGGCATCCTGAGGTTTAT–3′) and Reverse H-Cox1p2 (5′–TAAAGAAAGAACATAATGAAAATG–3′) primers [31] or Forward L-CO1p and Reverse HCOX1R (5′–AACAACAAACCAAGAATCATG–3′) primers [35]. Amplification and sequencing procedures were reported elsewhere in Héritier *et al.* [22], Meyer *et al.* [35] and Verneau *et al.* [51]. After inspection of chromatograms with SeqScape v2.5 software (Applied BioSystems, Waltham, MA, USA), sequences obtained in this study were aligned with all previously published COI sequences [5, 14, 22, 23, 35, 51], using Clustal W [47] implemented in MEGA6 [45]. Identical sequences were subsequently grouped into unique haplotypes, which were further analyzed with the help of MrBayes software [25].

Using Modeltest 3.06 [39], a GTR + I + Γ model was selected for running the Bayesian analysis. We defined three partitions according to codon positions 1, 2 and 3, and parameters for the selected GTR + I + Γ model were estimated independently for each partition (see Héritier *et al.* [22]). After running four chains of ten million generations each, which were sampled every 100 cycles, Bayesian posterior probabilities were estimated after removing the first 10,000 trees as the burn-in phase. The tree was rooted with sequences of two polystomes infecting amphibians, namely *Wetapolystoma almae* Gray, 1993 [18] and *Polystoma naevius* Caballero & Cerecero 1941 [4]. The final 50% majority consensus tree was then visualized with FigTree v.1.4.4 (http://tree.bio.ed.ac.uk/software/figtree/) and depicted as a radial tree to show phylogenetic relationships of all haplotypes and as a phenogram to depict phylogenetic relationships between all distinct species.

### Host and polystome species identification

Host species identity was mainly based on external morphological characters which permit turtle identification. When uncertainty was encountered for some specimens, the cytochrome b was sequenced and resulting nucleotide sequences were compared to sequence databases with BLAST (https://blast.ncbi.nlm.nih.gov/Blast.cgi) for species recognition. Concerning polystome species identity, whereas morphological characters are usually inadequate or not sufficiently informative for species recognition within a genus, morphometrics based on reproductive and fixing organs from mature worms are usually satisfactory for species determination [12]. However, morphometrics cannot be assessed from eggs collection. This is why polystome species delimitation was done here solely from COI DNA barcoding. Based on a cumulative error plot that was inferred from K2P COI distances within three polystomes, *i.e. P. oris*, *P. tunisiensis* and *P. euzeti*, Héritier *et al.* [22] found a large gap between intra and interspecific genetic divergences. Accordingly, they estimated that the probability of committing type I errors was less than 0.05 for co-specific specimens assuming an optimum threshold of 3.4% of genetic divergence. Therefore, species delimitation within polystomes was done according to the COI threshold designed by Héritier *et al.* [22].

## Results

**Figure 2:**
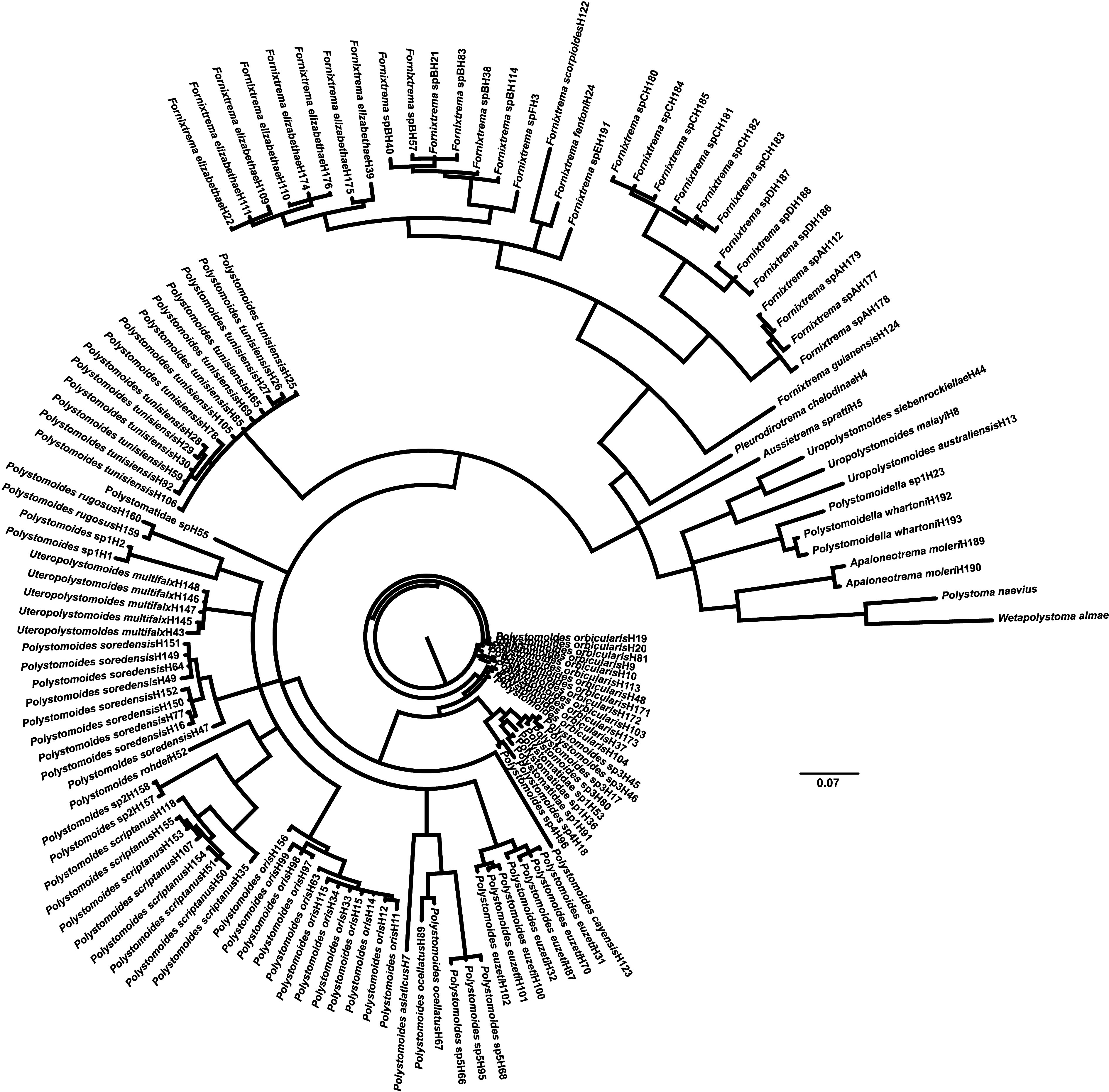
Radial tree depicting the phylogenetic relationships of the 134 discrete COI chelonian polystome haplotypes after Bayesian analysis.

**Figure 3:**
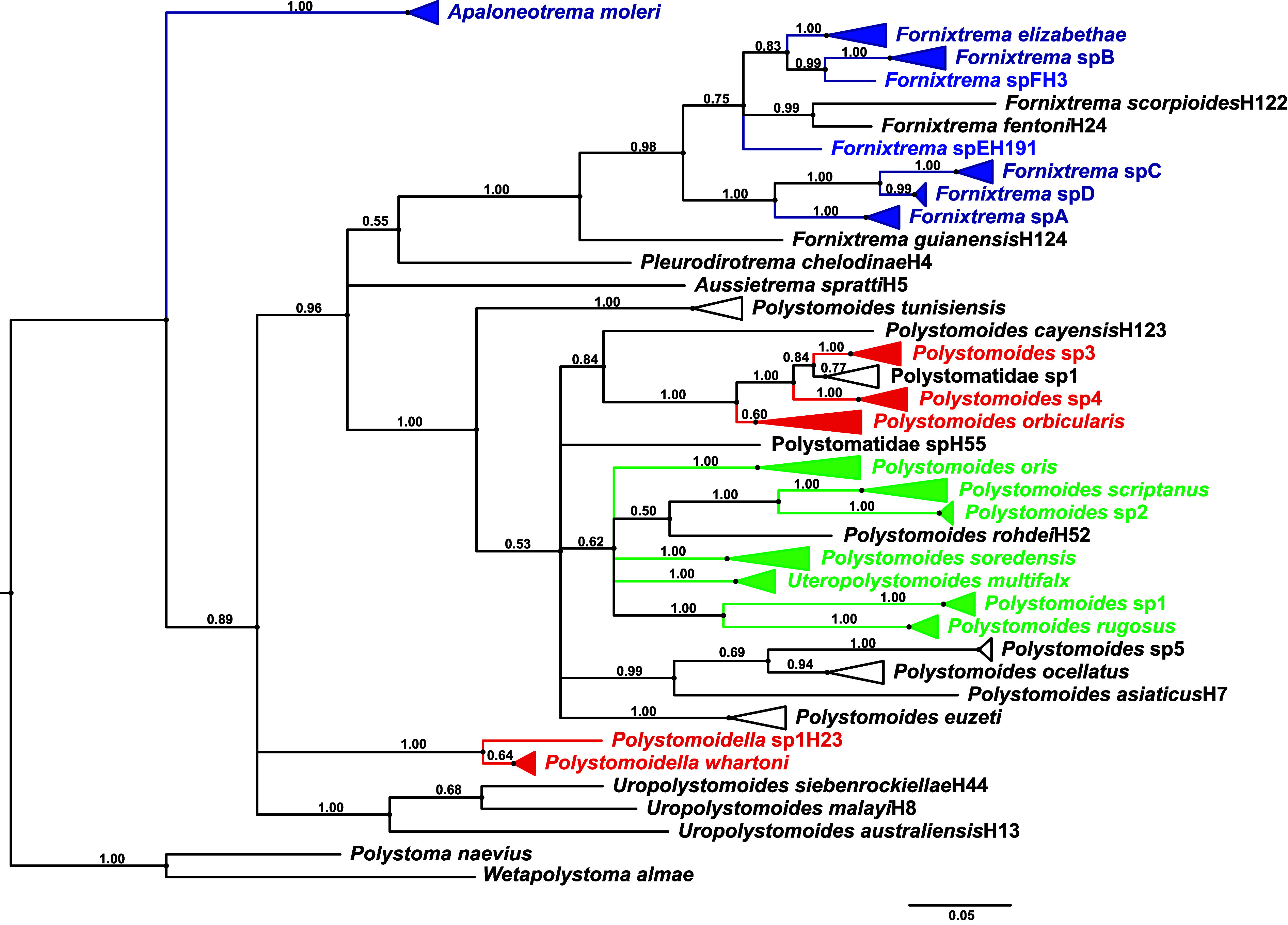
Phylogram of chelonian polystome species derived from the tree depicted in Figure 2 after collapsing haplotypes referring to the same species. Polystome species in blue refer to polystomes infecting the conjunctival sacs, in red the urinary bladder and in green the oral cavity of American turtles. Polystomatidae sp1 and sp2, which also infect American turtles, were not highlighted as the infection site was not determined.

The American freshwater turtles that were investigated for polystomes included *A. ferox*, *A. spinifera*, *C. picta*, *C. serpentina*, *G. pseudogeographica*, *K. baurii*, *Ps. concinna*, *Ps. floridana*, *Ps. nelsoni*, *Ps. peninsularis*, *S. minor*, *S. odoratus* and *T. scripta*. Prevalence of infection is summarized in Table 2 and in [5] for each turtle species according to their location. A total of 63 new sequences (accession numbers PQ052824 to PQ052886 in GenBank) were generated in this study from polystome eggs and/or adults that were collected in 2015 and 2018 (Table 3), characterizing 27 distinct haplotypes. When adding them to sequences extracted from GenBank, the COI haplotypic diversity for chelonian polystomes segregated into 134 distinct haplotypes (see Online Resource) whose phylogenetic relationships are depicted in Figure 2. Based on that tree and the COI threshold defined by Héritier *et al.* [22], 37 chelonian polystomes could be considered, whose relationships are depicted in Figure 3 after collapsing haplotypes referring to the same species. In total, we identified 22 polystome species infecting native and/or non-native American freshwater turtles, *i.e.*, eight from the conjunctival sacs, five from the urinary bladder, seven from the oral cavity and two from undetermined sites of infection (Table 4). Among these species, 13 are still presumptively undescribed based on the COI threshold designed for polystomes.

**Table 2:** List of North American turtle species investigated for polystomes in 2015 and 2018 (for those not listed in [5]), with location, prevalence of infection, infection site of polystomes and total number of worms collected after dissection.

Host species	State	Year of collection	Location	Number of turtles infected/examined	Type of eggs	Number of dissected turtles	Infection site of polystomes	Number of worms collected
*Apalone spinifera*	North Carolina	2015	loc10NC	0/1		0		
*Chelydra serpentina*	New York	2015	loc1NY	0/9		0		
*C. serpentina*	North Carolina	2015	loc4NC	0/1		0		
*C. serpentina*	North Carolina	2015	loc6NC	1/1	Long	1	Conjunctival sacs	5
*C. serpentina*	North Carolina	2015	loc8NC	0/2		0		
*C. serpentina*	North Carolina	2015	loc9NC	0/1		0		
*C. serpentina*	North Carolina	2015	loc10NC	0/1		0		
*C. serpentina*	North Carolina	2015	loc11NC	0/1		0		
*C. serpentina*	Florida	2018	loc3Fl	0/3		0		
*C. serpentina*	Florida	2018	loc5Fl	0/1		0		
*C. serpentina*	Florida	2018	loc9Fl	1/2	Long	0		
*C. serpentina*	Florida	2018	loc12Fl	0/1		0		
*C. serpentina*	Florida	2018	loc16Fl	0/13		0		
*Chrysemys picta*	Connecticut	2015	loc1Co	2/2	Round	1	Oral cavity	2
*C. picta*	Connecticut	2015	loc2Co	1/11	Round	0		
*C. picta*	New York	2015	loc1NY	3/23	Round & long	3	Oral cavity conjunctival sacs	3-0-1
								0-2-0
*C. picta*	North Carolina	2015	loc4NC	0/2		0		
*C. picta*	North Carolina	2015	loc5NC	3/22	Round & long	2	Oral cavity urinary bladder conjunctival sacs	1-5
								0-4
								1-5
*C. picta*	North Carolina	2015	loc6NC	0/8		0		
*C. picta*	North Carolina	2015	loc7NC	0/1		0		
*C. picta*	North Carolina	2015	loc9NC	0/1		0		
*C. picta*	North Carolina	2015	loc12NC	0/1		0		
*C. picta*	North Carolina	2015	loc13NC	0/5		0		
*Chrysemys* sp.	New York	2015	loc1NY	0/1		0		
*Sternotherus minor*	Florida	2018	loc3Fl	0/25		0		
*S. minor*	Florida	2018	loc12Fl	0/1		0		
*Sternotherus odoratus*	Florida	2018	loc1Fl	0/1		0		
*S. odoratus*	Florida	2018	loc5Fl	0/1		0		
*Trachemys scripta*	New York	2015	loc1NY	0/1		1		0
*T. scripta*	North Carolina	2015	loc5NC	0/1		0		
*T. scripta*	North Carolina	2015	loc6NC	0/1		0		
*T. scripta*	North Carolina	2015	loc7NC	1/1	Round	0		
*T. scripta*	North Carolina	2015	loc8NC	2/7	Round	1	Oral cavity	8
*T. scripta*	North Carolina	2015	loc10NC	3/7	Round	2	Urinary bladder	1-1
*Kinosternon baurii*	North Carolina	2015	loc5NC	1/2	Long	1	Conjunctival sacs	1
*K. baurii*	North Carolina	2015	loc10NC	0/9		0		
*K. baurii*	North Carolina	2015	loc13NC	0/1		0		
*K. baurii*	North Carolina	2018	loc2NC	2/4	Round	2	Urinary bladder	8-32
*K. baurii*	North Carolina	2018	loc3NC	0/1		0		
*K. baurii*	Florida	2018	loc1Fl	5/16	Round & long	3	Urinary bladder	6-0
							Conjunctival sacs	0-7
*K. baurii*	Florida	2018	loc12Fl	0/2		0		
*K. baurii*	Florida	2018	loc15Fl	1/2	Long	0		
*K. baurii*	Florida	2018	loc16Fl	1/1	Long	1	Conjunctival sacs	7
*Pseudemys concinna*	North Carolina	2015	loc4NC	0/1		0		

**Table 3:** Polystome samples (worms and/or eggs) for which partial COI sequences were obtained in this study.

Parasite species	Parasite tissue	Parasite field number	Infection site	DNA number	Haplotype number	G.B.A. number	Host species	Host field number	Geographical area and location in parentheses	Year of sample collection
*Apaloneotrema moleri*	1 worm	PL180713A1	Conj. sacs	MiAG77	H189	PQ052824	*Apalone ferox*	RL180713E1	Florida (loc8Fl)	2018
*Apaloneotrema moleri*	1 worm	PL180712A1	Conj. sacs	MiAG13	H190	PQ052825	*Apalone ferox*	RL180712A1	Florida (loc17Fl)	2018
*Fornixtrema elizabethae*	1 egg	3B1	Conj. sacs	MiAE159	H176	PQ052826	*Chelydra serpentina*	RL180703B1	North Carolina (loc6NC)	2018
*Fornixtrema elizabethae*	1 worm	PL150729C1	Conj. sacs	MiAD816	H109	PQ052827	*Chrysemys picta*	RL150728C1	North Carolina (loc5NC)	2015
*Fornixtrema elizabethae*	1 worm	PL150729A1	Conj. sacs	MiAD820	H110	PQ052828	*Chrysemys picta*	RL150726A2	North Carolina (loc5NC)	2015
*Fornixtrema elizabethae*	1 egg	26A2	Conj. sacs	MiAD825	H110	PQ052829	*Chrysemys picta*	RL150726A2	North Carolina (loc5NC)	2015
*Fornixtrema elizabethae*	1 egg	26A2	Conj. sacs	MiAD826	H110	PQ052830	*Chrysemys picta*	RL150726A2	North Carolina (loc5NC)	2015
*Fornixtrema elizabethae*	1 egg	26A2	Conj. sacs	MiAD827	H110	PQ052831	*Chrysemys picta*	RL150726A2	North Carolina (loc5NC)	2015
*Fornixtrema elizabethae*	1 egg	26A2	Conj. sacs	MiAD828	H110	PQ052832	*Chrysemys picta*	RL150726A2	North Carolina (loc5NC)	2015
*Fornixtrema elizabethae*	1 worm	PL150719C1	Conj. sacs	MiAD817	H111	PQ052833	*Chrysemys picta*	RL150715I4	New York (loc1NY)	2015
*Fornixtrema elizabethae*	1 egg	15I4	Conj. sacs	MiAD834	H111	PQ052834	*Chrysemys picta*	RL150715I4	New York (loc1NY)	2015
*Fornixtrema elizabethae*	1 egg	3A6	Conj. sacs	MiAE144	H175	PQ052835	*Chrysemys picta*	RL180703A6	North Carolina (loc5NC)	2018
*Fornixtrema elizabethae*	1 egg	4B1	Conj. sacs	MiAE146	H176	PQ052836	*Chrysemys picta*	RL180704B1	North Carolina (loc5NC)	2018
*Fornixtrema elizabethae*	1 egg	4K5	Conj. sacs	MiAE113	H174	PQ052837	*Trachemys scripta*	RL180704K5	North Carolina (loc1NC)	2018
*Fornixtrema* spA	1 worm	PL150729F1	Conj. sacs	MiAD819	H112	PQ052838	*Chelydra serpentina*	RL150723A1	North Carolina (loc6NC)	2015
*Fornixtrema* spA	1 egg	23A1	Conj. sacs	MiAD829	H112	PQ052839	*Chelydra serpentina*	RL150723A1	North Carolina (loc6NC)	2015
*Fornixtrema* spA	1 egg	23A1	Conj. sacs	MiAD830	H112	PQ052840	*Chelydra serpentina*	RL150723A1	North Carolina (loc6NC)	2015
*Fornixtrema* spA	1 egg	23A1	Conj. sacs	MiAD831	H112	PQ052841	*Chelydra serpentina*	RL150723A1	North Carolina (loc6NC)	2015
*Fornixtrema* spA	1 egg	13C1	Conj. sacs	MiAE26	H178	PQ052842	*Trachemys scripta*	RL180713C1	Florida (loc1Fl)	2018
*Fornixtrema* spA	1 egg	13C1	Conj. sacs	MiAE27	H178	PQ052843	*Trachemys scripta*	RL180713C1	Florida (loc1Fl)	2018
*Fornixtrema* spA	1 egg	13C1	Conj. sacs	MiAE28	H178	PQ052844	*Trachemys scripta*	RL180713C1	Florida (loc1Fl)	2018
*Fornixtrema* spA	1 egg	13C1	Conj. sacs	MiAE29	H178	PQ052845	*Trachemys scripta*	RL180713C1	Florida (loc1Fl)	2018
*Fornixtrema* spA	1 egg	13C4	Conj. sacs	MiAE36	H178	PQ052846	*Trachemys scripta*	RL180713C4	Florida (loc1Fl)	2018
*Fornixtrema* spA	1 egg	9B7	Conj. sacs	MiAE13	H177	PQ052847	*Trachemys scripta*	RL180709B7	Florida (loc9Fl)	2018
*Fornixtrema* spA	1 egg	9B7	Conj. sacs	MiAE14	H177	PQ052848	*Trachemys scripta*	RL180709B7	Florida (loc9Fl)	2018
*Fornixtrema* spA	1 egg	9B7	Conj. sacs	MiAE15	H177	PQ052849	*Trachemys scripta*	RL180709B7	Florida (loc9Fl)	2018
*Fornixtrema* spA	1 egg	5J3	Conj. sacs	MiAE154	H179	PQ052850	*Trachemys scripta*	RL180705J3	North Carolina (loc1NC)	2018
*Fornixtrema* spC	1 egg	15D9	Conj. sacs	MiAE68	H184	PQ052851	*Pseudemys concinna*	RL180715D9	Florida (loc5Fl)	2018
*Fornixtrema* spC	1 worm	PL180712D1	Conj. sacs	MiAG85	H185	PQ052852	*Pseudemys nelsoni*	RL180712B1	Florida (loc1Fl)	2018
*Fornixtrema* spC	1 egg	15B1	Conj. sacs	MiAE93	H181	PQ052853	*Pseudemys peninsularis*	RL180715B1	Florida (loc5Fl)	2018
*Fornixtrema* spC	1 egg	15B1	Conj. sacs	MiAE94	H181	PQ052854	*Pseudemys peninsularis*	RL180715B1	Florida (loc5Fl)	2018
*Fornixtrema* spC	1 egg	15B1	Conj. sacs	MiAE92	H182	PQ052855	*Pseudemys peninsularis*	RL180715B1	Florida (loc5Fl)	2018
*Fornixtrema* spC	1 worm	PL180718D1	Conj. sacs	MiAG88	H180	PQ052856	*Pseudemys peninsularis*	RL180718B1	Florida (loc7Fl)	2018
*Fornixtrema* spD	1 worm	PL180719A1	Conj. sacs	MiAG89	H187	PQ052857	*Pseudemys concinna*	RL180719A1	Florida (loc3Fl)	2018
*Fornixtrema* spD	1 egg	15D9	Conj. sacs	MiAE67	H188	PQ052858	*Pseudemys concinna*	RL180715D9	Florida (loc5Fl)	2018
*Fornixtrema* spD	1 egg	15B1	Conj. sacs	MiAE91	H186	PQ052859	*Pseudemys peninsularis*	RL180715B1	Florida (loc5Fl)	2018
*Fornixtrema* spE	1 worm	PL180717I1	Conj. sacs	MiAG35	H191	PQ052860	*Kinosternon baurii*	RL180713A11	Florida (loc1Fl)	2018
*Fornixtrema* spE	1 worm	PL180717I2	Conj. sacs	MiAG36	H191	PQ052861	*Kinosternon baurii*	RL180713A11	Florida (loc1Fl)	2018
*Fornixtrema* spE	1 egg	12D2	Conj. sacs	MiAE70	H191	PQ052862	*Kinosternon baurii*	RL180712D2	Florida (loc1Fl)	2018
*Fornixtrema* spE	1 egg	13A8	Conj. sacs	MiAE74	H191	PQ052863	*Kinosternon baurii*	RL180713A8	Florida (loc1Fl)	2018
*Fornixtrema* spE	1 egg	13A8	Conj. sacs	MiAE75	H191	PQ052864	*Kinosternon baurii*	RL180713A8	Florida (loc1Fl)	2018
*Polystomoidella whartoni*	1 egg	13A3	Urin. bladder	MiAE72	H193	PQ052865	*Kinosternon baurii*	RL180713A3	Florida (loc1Fl)	2018
*Polystomoidella whartoni*	1 egg	13A3	Urin. bladder	MiAE73	H193	PQ052866	*Kinosternon baurii*	RL180713A3	Florida (loc1Fl)	2018
*Polystomoidella whartoni*	1 worm	PL180707N4	Urin. bladder	MiAG42	H192	PQ052867	*Kinosternon baurii*	RL180705E2	North Carolina (loc2NC)	2018
*Polystomoidella whartoni*	1 worm	PL180707M10	Urin. bladder	MiAG43	H192	PQ052868	*Kinosternon baurii*	RL180705E3	North Carolina (loc2NC)	2018
*Polystomoidella whartoni*	1 worm	PL180707M9	Urin. bladder	MiAG44	H192	PQ052869	*Kinosternon baurii*	RL180705E3	North Carolina (loc2NC)	2018
*Polystomoidella whartoni*	1 worm	PL180707N7	Urin. bladder	MiAG45	H192	PQ052870	*Kinosternon baurii*	RL180705E2	North Carolina (loc2NC)	2018
*Polystomoidella whartoni*	1 egg	5E2	Urin. bladder	MiAE164	H192	PQ052871	*Kinosternon baurii*	RL180705E2	North Carolina (loc2NC)	2018
*Polystomoidella whartoni*	1 egg	5E2	Urin. bladder	MiAE165	H192	PQ052872	*Kinosternon baurii*	RL180705E2	North Carolina (loc2NC)	2018
*Polystomoidella whartoni*	1 egg	5E2	Urin. bladder	MiAE166	H192	PQ052873	*Kinosternon baurii*	RL180705E2	North Carolina (loc2NC)	2018
*Polystomoidella whartoni*	1 egg	5E2	Urin. bladder	MiAE167	H192	PQ052874	*Kinosternon baurii*	RL180705E2	North Carolina (loc2NC)	2018
*Polystomoidella whartoni*	1 egg	5E2	Urin. bladder	MiAE168	H192	PQ052875	*Kinosternon baurii*	RL180705E2	North Carolina (loc2NC)	2018
*Polystomoidella whartoni*	1 egg	5E2	Urin. bladder	MiAE169	H192	PQ052876	*Kinosternon baurii*	RL180705E2	North Carolina (loc2NC)	2018
*Polystomoidella whartoni*	1 egg	5E2	Urin. bladder	MiAE170	H192	PQ052877	*Kinosternon baurii*	RL180705E2	North Carolina (loc2NC)	2018
*Polystomoidella whartoni*	1 egg	5E2	Urin. bladder	MiAE171	H192	PQ052878	*Kinosternon baurii*	RL180705E2	North Carolina (loc2NC)	2018
*Polystomoides orbicularis*	1 egg	5A1	Urin. bladder	MiAE126	H173	PQ052879	*Trachemys scripta*	RL180705A1	North Carolina (loc6NC)	2018
*Polystomoides orbicularis*	1 egg	13C13	Urin. bladder	MiAE46	H172	PQ052880	*Trachemys scripta*	RL180713C13	Florida (loc1Fl)	2018
*Polystomoides orbicularis*	1 worm	PL180717C1	Urin. bladder	MiAG8	H171	PQ052881	*Trachemys scripta*	RL180709D1	Florida (loc3Fl)	2018
*Polystomoides orbicularis*	1 egg	9D1	Urin. bladder	MiAE16	H171	PQ052882	*Trachemys scripta*	RL180709D1	Florida (loc3Fl)	2018
*Polystomoides orbicularis*	1 worm	PL180716G1	Urin. bladder	MiAG6	H48	PQ052883	*Trachemys scripta*	RL180709B1	Florida (loc9Fl)	2018
*Polystomoides orbicularis*	1 egg	9B1	Urin. bladder	MiAE1	H48	PQ052884	*Trachemys scripta*	RL180709B1	Florida (loc9Fl)	2018
*Polystomoides orbicularis*	1 egg	9B3	Urin. bladder	MiAE5	H48	PQ052885	*Trachemys scripta*	RL180709B3	Florida (loc9Fl)	2018
*Polystomoides orbicularis*	1 egg	9B4	Urin. bladder	MiAE9	H48	PQ052886	*Trachemys scripta*	RL180709B4	Florida (loc9Fl)	2018

Abbreviations used: G.B.A. = GenBank Accession; Conj. sacs = Conjunctival sacs; Urin. bladder = Urinary bladder.

Note. GPS coordinates of geographical areas and locations are specified in Table 1.

**Table 4:** Polystome species and haplotypic diversity among American (*Apalone ferox*, *A. spinifera*, *Chelydra serpentina, Chrysemys picta*, *Graptemys pseudogeographica*, *Kinosternon baurii*, *Pseudemys concinna*, *P. floridana*, *P. nelsoni*, *P. peninsularis* and *Trachemys scripta*) and Mediterranean (*Emys orbicularis* and *Mauremys leprosa*) turtle species across natural North American and Mediterranean freshwater environments, as well as in outdoor turtle enclosures.

Host species	Polystome species	Infection site	COI Haplotype	Geographical area and location in brackets
American turtles				
*Apalone ferox*	*Apaloneotrema moleri*	Conjunctival sacs	H189	Florida (loc8Fl)
			H190	Florida (loc17Fl)
	*Polystomoides rugosus*	Oral cavity	H159	Florida (loc8Fl)
			H160	Florida (loc17Fl)
*Apalone spinifera*	*Polystomoides* sp1	Oral cavity	H1	Indiana
			H2	France (loc22Fc)
*Chelydra serpentina*	*Fornixtrema elizabethae*	Conjunctival sacs	H176	North Carolina (loc6NC)
	*Fornixtrema* spA	Conjunctival sacs	H112	North Carolina (loc6NC)
	*Fornixtrema* spF	Conjunctival sacs	H3	Nebraska
*Chrysemys picta*	*Fornixtrema elizabethae*	Conjunctival sacs	H22	Connecticut (loc1Co)
			H109	North Carolina (loc5NC)
			H110	North Carolina (loc5NC)
			H111	New York (loc1NY)
			H175	North Carolina (loc5NC)
			H176	North Carolina (loc5NC)
	*Polystomoides orbicularis*	Urinary bladder	H9	Indiana
			H10	Indiana
			H103	North Carolina (loc5NC)
	*Polystomoides oris*	Oral cavity	H11	Indiana
			H12	Indiana
			H97	Connecticut (loc1Co) & New York (loc1NY)
			H98	New York (loc1NY)
			H99	North Carolina (loc5NC)
			H156	North Carolina (loc2NC)
*Graptemys*	*Fornixtrema* spB	Conjunctival sacs	H21	Indiana
*pseudogeographica*				
*Kinosternon baurii*	*Fornixtrema elizabethae*	Conjunctival sacs	H175	North Carolina (loc5NC)
	*Fornixtrema* spE	Conjunctival sacs	H191	Florida (loc1Fl)
	*Polystomoidella whartoni*	Urinary bladder	H192	North Carolina (loc2NC)
			H193	Florida (loc1Fl)
	*Polystomoidella* sp1	Urinary bladder	H23	Florida (loc15Fl)
*Pseudemys concinna*	*Fornixtrema* spC	Conjunctival sacs	H184	Florida (loc5Fl)
	*Fornixtrema* spD	Conjunctival sacs	H187	Florida (loc3Fl)
			H188	Florida (loc5Fl)
	*Uteropolystomoides multifalx*	Oral cavity	H145	Florida (loc2Fl)
			H146	Florida (loc2Fl & loc5Fl)
			H147	Florida (loc2Fl)
			H148	Florida (loc3Fl & loc5Fl)
*Pseudemys floridana*	*Uteropolystomoides multifalx*	Oral cavity	H145	Florida (loc1Fl)
			H147	Florida (loc1Fl)
*Pseudemys nelsoni*	*Fornixtrema* spC	Conjunctival sacs	H185	Florida (loc1Fl)
	*Uteropolystomoides multifalx*	Oral cavity	H43	Florida (loc14Fl)
*Pseudemys peninsularis*	*Fornixtrema* spC	Conjunctival sacs	H180	Florida (loc7Fl)
			H181	Florida (loc5Fl)
			H182	Florida (loc5Fl)
			H183	Florida (loc5Fl)
	*Fornixtrema* spD	Conjunctival sacs	H186	Florida (loc5Fl)
	*Polystomoides scriptanus*	Oral cavity	H153	Florida (loc5Fl)
*Trachemys scripta*	Fornixtrema elizabethae*	Conjunctival sacs	H174	North Carolina (loc1NC)
	*Fornixtrema* spA*	Conjunctival sacs	H177	Florida (loc9Fl)
			H178	Florida (loc1Fl)
			H179	North Carolina (loc1NC)
	*Fornixtrema* spB	Conjunctival sacs	H21	France (loc18Fc)
			H38	France (loc22Fc)
			H114	France (loc22Fc)
	Polystomoides orbicularis*	Urinary bladder	H20	France (loc19Fc & loc22Fc)
			H37	France (loc20Fc)
			H48	Florida (loc9Fl & loc15Fl)
			H81	France (loc19Fc)
			H104	North Carolina (loc3NC)
			H171	Florida (loc3Fl)
			H172	Florida (loc1Fl)
			H173	North Carolina (loc6NC)
	*Polystomoides* sp3*	Urinary bladder	H17	France (loc22Fc)
			H45	Indiana
			H46	Kansas
	*Polystomoides* sp4	Urinary bladder	H18	France (loc18Fc & loc20Fc)
	*Polystomoides oris*	Oral cavity	H15	France (loc22Fc)
			H115	France (loc3Fc)
	Polystomoides scriptanus*	Oral cavity	H35	France (loc22Fc)
			H50	Florida (loc15Fl)
			H51	Florida (loc15Fl)
			H107	North Carolina (loc8NC)
			H118	North Carolina (loc7NC & loc8NC)
			H153	Florida (loc1Fl)
			H154	Florida (loc13Fl)
			H155	Florida (loc13Fl)
	Polystomoides soredensis*	Oral cavity	H16	France (loc19Fc)
			H47	Indiana
			H49	Maine
			H64	North Carolina (loc1NC, loc3NC & loc4NC) & France (loc10Fc)
			H77	France (loc20Fc)
			H149	North Carolina (loc1NC, loc4NC & loc6NC)
			H150	North Carolina (loc1NC)
			H151	Florida (loc1Fl)
			H152	North Carolina (loc3NC)
	*Polystomoides* sp2*	Oral cavity	H157	North Carolina (loc1NC)
			H158	North Carolina (loc1NC)
	Polystomatidae sp1	Unknown	H91	France (loc4Fc & loc18Fc)
	Polystomatidae sp2	Unknown	H55	France (loc22Fc)
Mediterranean turtles				
*Emys orbicularis*	*Fornixtrema elizabethae*	Conjunctival sacs	H22	France (loc22Fc)
	*Fornixtrema* spB	Conjunctival sacs	H21	France (loc16Fc, loc17Fc &22Fc)
	*Polystomoides* sp3	Urinary bladder	H17	France (loc3Fc & loc22Fc)
	*Polystomoides* sp4	Urinary bladder	H18	France (loc16Fc & loc24Fc)
			H96	France (loc5Fc & loc24Fc)
	*Polystomoides ocellatus*	Oral cavity	H67	France (loc5Fc, loc24Fc, loc25Fc & loc26Fc) & Spain (Estanya & La Alfranca)
			H89	Algeria (El Kala)
	*Polystomoides oris*	Oral cavity	H14	France (loc6Fc & loc22Fc)
			H15	France (loc1Fc, loc2Fc, loc14Fc, loc22Fc, loc24Fc & loc26Fc) & Spain (Berroya & Sastoya)
			H63	France (loc8Fc)
	*Polystomoides soredensis*	Oral cavity	H16	France (loc22Fc)
	*Polystomoides* sp5	Oral cavity	H66	Spain (Arielz, Bardenas, Berroya, Caparroso, Gallipienzo, La Alfranca, Murillo El Fruto, Pina de Ebro, Sastoya & Ujué)
			H68	France (loc7Fc & loc9Fc)
			H95	Spain (Porriño & artificial site Betanzos)
	Polystomatidae sp1	Unknown	H36	France (loc8Fc, loc13Fc & Loc15Fc)
			H53	France (loc 11Fc & loc12Fc)
			H91	France (loc18Fc)
*Mauremys leprosa*	*Fornixtrema elizabethae*	Conjunctival sacs	H22	France (loc22Fc)
			H39	France (loc22Fc)
	*Fornixtrema* spB	Conjunctival sacs	H21	France (loc17Fc, loc18Fc, loc19Fc, loc22Fc & loc23Fc)
			H38	France (loc22Fc)
			H40	France (loc22Fc)
			H57	France (loc17Fc)
			H83	France (loc23Fc)
	*Polystomoides euzeti*	Urinary bladder	H31	Spain (Merdanc & Orlina) & Algeria (El Amra, Oued Rhiou & Rouina)
			H32	Spain (Orlina) & France (loc21Fc & loc22Fc)
			H70	Algeria (Réghaïa)
			H87	Algeria (Rouina)
			H100	Morocco (Ahl Souss)
			H101	Morocco (Ben Ahmed)
			H102	Morocco (Ben Ahmed)
			H116	France (loc6Fc)
	*Polystomoides orbicularis*	Urinary bladder	H19	France (loc22Fc) & Spain (Orlina)
			H20	France (loc19Fc & loc22Fc)
			H37	France (loc19Fc, loc20Fc & loc22Fc)
	*Polystomoides* sp3	Urinary bladder	H17	Spain (Anyet)
			H80	Spain (Anyet)
	*Polystomoides* sp4	Urinary bladder	H18	France (loc23Fc)
	*Polystomoides oris*	Oral cavity	H14	France (loc22Fc)
			H15	France (loc22Fc)
			H33	France (loc22Fc)
			H34	France (loc22Fc)
	*Polystomoides scriptanus*	Oral cavity	H35	France (loc22Fc)
	*Polystomoides soredensis*	Oral cavity	H16	France (loc19Fc & loc20Fc)
			H77	France (loc20Fc)
	*Polystomoides tunisiensis*	Oral cavity	H25	Algeria (Oued Rhiou & Rouina)
			H26	France (loc19Fc)
			H27	France (loc22Fc)
			H28	France (loc22Fc)
			H29	France (loc22Fc)
			H30	France (loc21Fc)
			H59	France (loc3Fc) & Spain (Anyet, La Alfranca, Merdanc & Orlina)
			H65	Morocco (Ben Ahmed)
			H69	Algeria (Rouina)
			H78	Spain (Anyet & Orlina)
			H82	Spain (Anyet, Merdanc & Orlina)
			H85	Algeria (El Amra)
			H105	Algeria (El Kala)
			H106	Algeria (Constantine)
	Polystomatidae sp1	Unknown	H36	France (loc22Fc)

Note. GPS coordinates for American and French localities are specified in Table 1, while GPS coordinates for other localities (Spain, Morocco and Algeria) can be retrieved in Meyer *et al.* [35] and Héritier *et al.* [22]. Polystomatidae sp1 and Polystomatidae sp2 could not be classified at the genus level as no adult worms were extracted from infected turtles. Underlined polystome species refer to polystomes that are shared between *T. s. elegans* and the two native freshwater turtles *E. orbicularis* and *M. leprosa* in Southern Europe. Polystome species labeled with an asterisk refer to polystomes found within American populations of *T. s. elegans*.

The polystome diversity of *T. s. elegans* currently stands at 12 species, considering that host sampling was conducted across freshwater ecosystems in the United States, but also in France and Spain where the red-eared slider was introduced. These species were *Fornixtrema elizabethae* (Platt, 2000 [38]), *Fornixtrema* spA, *Fornixtrema* spB, *P. orbicularis*, *P. oris*, *Polystomoides scriptanus* Héritier *et al.*, 2018 [23], *Polystomoides soredensis* Héritier *et al.*, 2018 [23], *Polystomoides* sp2, *Polystomoides* sp3, *Polystomoides* sp4, Polystomatidae sp1 and Polystomatidae sp2. Additionally, there were two polystomes associated with *A. ferox*, namely *Apaloneotrema moleri* (Du Preez & Morrison 2012 [13]) and *Polystomoides rugosus* (MacCallum, 1918 [32]), a single polystome associated with *A. spinifera*, namely *Polystomoides* sp1, three polystomes associated with *C. serpentina*, namely *F. elizabethae*, *Fornixtrema* spA and *Fornixtrema* spF, three polystomes associated with *C. picta,* namely *F. elizabethae*, *P. orbicularis* and *P. oris*, a single polystome with *G. pseudogeographica*, namely *Fornixtrema* spB, three polystomes associated with *K. baurii*, namely *Fornixtrema* spE, *Polystomoidella whartoni* Price 1939 [41] and *Polystomoidella* sp1, three polystomes with *Ps. concinna*, namely *Fornixtrema* spC, *Fornixtrema* spD and *Uteropolystomoides multifalx* (Stunkard, 1924 [44]), a single polystome with *Ps. floridana*, namely *U. multifalx*, two polystomes with *Ps. nelsoni*, namely *Fornixtrema* spC and *U. multifalx*, and three polystomes with *Ps. peninsularis*, namely *Fornixtrema* spC, *Fornixtrema* spD and *P. scriptanus* (Table 4).

## Discussion

### The ecological role of *T. s. elegans* in polystome spreading across European freshwater ecosystems potentially showing a spillover effect

Regarding the polystome diversity within *T. s. elegans* across European natural freshwater ecosystems and in outdoor turtle enclosures – nine species in total – it should be noted that all but one, Polystomatidae sp2 of the red-eared slider, were shared with *M. leprosa* and *E. orbicularis* in their native ranges (underlined polystome species in Table 4). Another one, *i.e.*, *F. elizabethae*, was also shared between *T. s. elegans* and *M. leprosa* but never reported from *T. s. elegans* in European freshwater environments. Considering that these parasites originated from American turtles, Héritier *et al.* [22] and Meyer *et al.* [35] hypothesized that host switching may have occurred from *T. s. elegans* to both European turtles, either in the wild, when turtles were found sympatrically in the same habitats, or following the release or translocation of native turtles after they became infected in outdoor turtle enclosures. This conclusion was also supported by the lack of *T. s. elegans* across freshwater ecosystems of Algeria and Morocco and the absence of its polystomes through *M. leprosa* and *E. orbicularis* in these environments. Héritier *et al.* [22] and Meyer *et al.* [35] thus considered that *T. s. elegans* could serve as a carrier of alien parasites in natural environments. Conversely, the native polystomes of *M. leprosa*, namely *P. euzeti* and *P. tunisiensis*, and those of *E. orbicularis*, namely *P. ocellatus*, were never reported within *T. s. elegans*, whether in natural environments or in confined environments such as outdoor turtle enclosures. Therefore, *T. s. elegans* may serve as a reservoir host for spillover of non-indigenous polystomes across turtles in European freshwater environments, but does not appear to act as a new reservoir host for native polystomes in the same environments.

As a result, this raises numerous questions about the immune response of the red-eared slider to polystomes. How can we explain the susceptibility of *T. s. elegans* to its own polystomes, as well as to polystomes of *C. picta* and *G. pseudogeographica* for instance, while it appears to be resistant to the polystomes of *E. orbicularis*, which belongs to the same turtle family Emydidae, and to polystomes of *M. leprosa*? Polystomes found in American turtles could be less susceptible to immune defenses than are polystomes inhabiting European turtles, or simply better competitors. Regardless of the reason, *T. s. elegans* may then act as a sink reservoir host for European polystomes contributing to a dilution effect in their dynamics (see [6, 15]). This would explain the paucity of native polystomes, *i.e.*, *P. euzeti* and *P. tunisiensis* in *M. leprosa* and *P. ocellatus* in *E. orbicularis*. Ultimately, if *T. s. elegans* serves as a source of non-native polystomes and as a sink for native polystomes in freshwater environments, all three native polystomes could quickly go extinct, at least across European natural environments (see also [22, 35]). While experimental infestations have been completed with polystomes of amphibians [1, 11, 26, 29] and turtles [37], cross experimental infestations with native and non-native parasites could be used to study the immune response of the host [27, 48, 49], with respect to relative competitive ability and interactions between polystomes [2, 48].

### The ecological role of *T. s. elegans* in polystome transmission across American freshwater ecosystems potentially showing a spillback effect

While nine polystomes were recorded from either captive or feral populations of *T. s. elegans* across European freshwater ecosystems, only seven species were documented within red-eared sliders across American wetland environments (polystome species with an asterisk in Table 4). Among these, three species, *i.e.*, *P. scriptanus*, *P. soredensis* and *Polystomoides* sp2 were reported from the pharyngeal cavity of their hosts. *Polystomoides scriptanus* occurred in Florida and North Carolina, *P. soredensis* occurred in Florida, Indiana, Maine and North Carolina, whereas *Polystomoides* sp2 occurred only in North Carolina. Despite the occurrence of *P. scriptanus* in *M. leprosa* from outdoor turtle enclosures and the occurrence of *P. soredensis* in *M. leprosa* and *E. orbicularis* from outdoor turtle enclosures and across European natural environments, *T. s. elegans* was considered the original host species for both polystomes by Héritier *et al.* [23]. Because *M. leprosa* and *E. orbicularis* are not found in American wetlands, Héritier *et al.* [23] considered that it was very unlikely that European turtles serve as a carrier for polystomes to the United States. One may therefore question the origin of these two distinct polystomes as well as the origin of *Polystomoides* sp2 in the pharyngeal cavity of the same host species across American freshwater environments. While *T. s. elegans* has been introduced globally into developed countries where young turtles were sold as pets, its current distribution in the United States also extends beyond its native range [3, 50]. The presence of *T. s. elegans* in Florida, Maine and North Carolina is also recognized as a consequence of the release of pet turtles that have become established in feral populations, while Indiana and Kansas are part of their native distribution. Considering that selection over evolutionary timescales should have led to a single polystome within each ecological niche in the host, we hypothesize that *P. soredensis* is a true native polystome of *T. s. elegans* as it is the single species infecting the pharyngeal cavity of the red-eared slider in the state of Indiana. We also assume that *Polystomoides* sp2 and *P. scriptanus* may have colonized *T. s. elegans* from other American turtle species. Even though the host has not yet been identified for *Polystomoides* sp2, it could be *Ps. peninsularis* for *P. scriptanus*, *Ps. peninsularis* being indeed the single American turtle species also infected by this parasite. Besides these three polystomes that were recorded from *T. s. elegans* across American wetland environments, *P. oris* was also reported from *T. s. elegans*, however only from European freshwater environments. Because *P. oris* was described early by Paul [36] from *C. picta* in the United States, it is very likely that *P. oris* infected *T. s. elegans* in this country from *C. picta*.

Two other polystomes, *i.e.*, *P. orbicularis* and *Polystomoides* sp3, were recorded within red-eared sliders across American wetland environments, both infecting the urinary bladder of their host. Concerning *Polystomoides* sp3, which has not yet been described, it was only found in Indiana and Kansas, which both correspond to the native range of the red-eared slider. This species may therefore represent a tissue-specific polystome found in the urinary bladder of its host. In the same way as discussed above for *P. soredensis*, we may hypothesize that this innominate species is also a true native polystome for the red-eared slider. For *P. orbicularis*, while it was reported from *T. s. elegans* in Florida and North Carolina, which are two areas where the red-eared slider was introduced, it was not recorded in Indiana and Kansas. Moreover, *P. orbicularis* was documented within *C. picta* in Indiana and North Carolina. Because the painted turtle has been recognized as the original host of *P. orbicularis*, the occurrence of this parasite in the urinary bladder of *T. s. elegans* likely reflects a switch from painted turtles to red-eared sliders across American freshwater environments.

Finally, three polystomes, *i.e.*, *F. elizabethae*, *Fornixtrema* spA and *Fornixtrema* spB were recorded within red-eared sliders across American wetland environments, all of them infecting the conjunctival sacs of their host. Since *F. elizabethae* was described from *C. picta* in Michigan and reported from the same host species in Wisconsin and Indiana, on the one hand [38], and because *Fornixtrema* spB was reported from *G. pseudogeographica* in Indiana, on the other (Platt, unpublished observations), it is likely that these two species switched from these two hosts to *T. s. elegans*. If we rely on this hypothesis*, F. elizabethae* may also have switched to *C. serpentina*. Finally, as *Fornixtrema* spA was also reported from *C. serpentina*, a switch from *C. serpentina* to *T. s. elegans* is also plausible.

As a consequence, *P. soredensis* and *Polystomoides* sp3 may represent native polystomes for *T. s. elegans*, infecting the pharyngeal cavity and the urinary bladder of their host, respectively, whereas *P. scriptanus, Polystomoides* sp2 and *P. oris* from the pharyngeal cavity, *P. orbicularis* from the urinary bladder and *F. elizabethae*, *Fornixtrema* spA and *Fornixtrema* spB from the conjunctival sacs may represent non-native polystomes for *T. s. elegans*. Therefore, if the red-eared slider acts as a reservoir host for spillover of polystomes in non-American freshwater environments, thus enhancing exotic parasite spreading (see above), given our data, *T. s. elegans* could act as a reservoir host for spillback of polystomes in American freshwater environments, thus increasing native parasite transmission in the wild.

## Conclusion

Our results on the origin and distribution of polystomes in American and European freshwater environments still raise numerous questions, including (i) the extent of polystome diversity within *T. s. elegans* in natural environments in the United States, (ii) which species of American turtles served as parasite donors before polystomes were dispersed through red-eared sliders acting as reservoir hosts across European wetland ecosystems, and (iii) because *T. s. elegans* acts as a reservoir host for spillover of polystomes among European freshwater turtles and as a reservoir host for spillback of polystomes among American freshwater turtles, what are the genetic determinants underlying host specificity? To answer these questions, infection experiments and parasitological surveys in areas where turtle diversity is the highest and where *T. s. elegans* also occurs with other native species are needed. We therefore plan to conduct cross-infection experiments with native and non-native polystomes which will constitute the first stage to understand how host-specific polystomes may become generalists in this existing turtle system and help better understand the consequences of parasite invasions driven by an invasive turtle species. The absence of *P. oris*, for instance, within American red-eared sliders while it occurs within painted turtles in the states of Connecticut, Indiana, New York and North Carolina as well as within red-eared sliders in outdoor turtle enclosures in France and within native European turtle species across natural environments, still remains unclear.

## Supplementary material

The supplementary material of this article is available at https://www.parasite-journal.org/10.1051/parasite/2025022/olm. *Online Resource 1*. COI haplotype diversity of polystomes sampled from turtles in aquatic natural environments and/or confined areas. Lines in purple indicate parasite specimens collected in outdoor turtle enclosures.
